# Estrogen rescues heart failure through estrogen receptor Beta activation

**DOI:** 10.1186/s13293-018-0206-6

**Published:** 2018-10-30

**Authors:** Andrea Iorga, Soban Umar, Gregoire Ruffenach, Laila Aryan, Jingyuan Li, Salil Sharma, Negar Motayagheni, Rangarajan D. Nadadur, Jean C. Bopassa, Mansoureh Eghbali

**Affiliations:** 1 Department of Anesthesiology, Division of Molecular Medicine, Cardiovascular Research Laboratories, David Geffen School of Medicine at University of California Los Angeles, Los Angeles, CA 90095 USA; 2 Present address: Department of Medicine, Division of Gastroenterology/Liver, Keck School of Medicine of the University of Southern California, Los Angeles, CA 90033 USA; 3 Present Address: Wake Forest Institute for Regenerative Medicine, Wake Forest University, Winston-Salem, NC 27109, USA; 4 Present address: Department of Physiology, University of Texas Health Science Center, San Antonio, TX 78229 USA

**Keywords:** Estrogen, Heart failure, Angiogenesis, Fibrosis, Estrogen receptor Beta, Estrogen receptor alpha

## Abstract

**Background:**

Recently, we showed that exogenous treatment with estrogen (E2) rescues pre-existing advanced heart failure (HF) in mice. Since most of the biological actions of E2 are mediated through the classical estrogen receptors alpha (ERα) and/or beta (ERβ), and both these receptors are present in the heart, we examined the role of ERα and ERβ in the rescue action of E2 against HF.

**Methods:**

Severe HF was induced in male mice by transverse aortic constriction-induced pressure overload. Once the ejection fraction (EF) reached ~ 35%, mice were treated with selective agonists for ERα (PPT, 850 μg/kg/day), ERβ (DPN, 850 μg/kg/day), or E2 (30 μg/kg/day) together with an ERβ-antagonist (PHTPP, 850 μg/kg/day) for 10 days.

**Results:**

EF of HF mice was significantly improved to 45.3 ± 2.1% with diarylpropionitrile (DPN) treatment, but not with PPT (31.1 ± 2.3%). E2 failed to rescue HF in the presence of PHTPP, as there was no significant improvement in the EF at the end of the 10-day treatment (32.5 ± 5.2%). The improvement of heart function in HF mice treated with ERβ agonist DPN was also associated with reduced cardiac fibrosis and increased cardiac angiogenesis, while the ERα agonist PPT had no significant effect on either cardiac fibrosis or angiogenesis. Furthermore, DPN improved hemodynamic parameters in HF mice, whereas PPT had no significant effect.

**Conclusions:**

E2 treatment rescues pre-existing severe HF mainly through ERβ. Rescue of HF by ERβ activation is also associated with stimulation of cardiac angiogenesis, suppression of fibrosis, and restoration of hemodynamic parameters.

## Background

Heart hypertrophy is usually triggered by external stressors. While it is presumed to be compensatory at the beginning, it often progresses to chronic heart failure (HF) if the external stimulus persists. HF is characterized by the progressive loss of contractility of the myocardium, leading to diminished cardiac output [1]. Female sex is a significant independent predictor of survival in patients with congestive HF [2], and women with advanced HF appear to have better survival outcome than men [3]. Estrogen has been shown to confer cardioprotection in experimental models of HF [4, 5]. Pre-treatment with E2 reduced the extent of pressure overload-induced hypertrophy and attenuated the deterioration of left ventricular (LV) systolic function and contractility [6]. We have also shown recently that E2 is even able to rescue pre-existing HF [5]. Estrogen mainly acts through classical estrogen receptor alpha (ERα) and/or estrogen receptor beta (ERβ), and both receptors are present in the heart [7, 8]. The beneficial role of ERβ in cardioprotection has been highlighted in the recent years [4, 9, 10]. Female mice lacking ERβ are less protected against myocardial ischemia/reperfusion injury compared to wild-type female mice. Furthermore, ERβ knockout mice have abnormal vascular function and hypertension, increased mortality, and aggravated heart failure [11]. Activation of ERβ has also been shown to confer the anti-hypertrophic effects of E2 [10].

Increased cardiac angiogenesis is a key event in maintaining LV function during adaptive hypertrophy induced by pressure overload; however, the imbalance between cardiac growth and neoangiogenesis eventually leads to the transition from compensated heart hypertrophy to HF [12]. Estrogen has previously been shown to be pro-angiogenic in various tissues [2–4, 11–15]. Recently, we have shown that E2 stimulates angiogenesis in the heart in the LV failure model induced by pressure overload as well as in right ventricular failure induced by pulmonary hypertension [4, 5].

Cardiac remodeling in HF is known to be associated with fibrosis, which leads to stiffening of the cardiac muscle and myocyte electrical uncoupling, thus impeding both contraction and relaxation of the heart. E2 is known to reduce adverse extracellular matrix remodeling in LV hypertrophy and failure by decreasing collagen deposition and metalloproteinase expression [16]. We have recently shown that E2-induced rescue of HF is associated with the reversal of LV fibrosis as well as downregulation of pro-fibrotic genes such as collagen I/III, transforming growth factor-β1 (TGF-β1), fibrosin I, and lysil oxidase (LOX) to levels similar to controls [5].

Although various recent studies have investigated the role of sex differences and E2 in the rescue of advanced HF [5, 17, 18], the precise contribution of the major estrogen receptors in the E2-induced rescue of HF has not been fully elucidated. Here, we investigated the role of ERα and ERβ in the rescue of HF by E2. We used the well-established transverse aortic constriction (TAC) model to induce HF in mice and show that short-term therapy with an ERβ-agonist, but not ERα-agonist, starting at a late stage of HF reverses the myocardial contractile dysfunction by significantly improving EF from 33 to ~ 45%. This improvement of myocardial contractility by ERβ was associated with increased cardiac angiogenesis, reduced cardiac fibrosis together with downregulation of pro-fibrotic gene expression and decreased hypertrophy.

## Methods

### Animals

Wild-type male CD-1 mice (3–4 months old) from Charles River were used for the study. All protocols received institutional review and committee approval from the Division of Laboratory Animal Medicine (DLAM) at UCLA. Mice were housed at DLAM with ad libitum regular chow and water access.

### Anesthesia and analgesia

For sham or TAC surgery, as well as terminal catheterization and euthanasia, mice were anesthetized with ketamine (100 mg/kg, intraperitoneally) and xylazine (10 mg/kg, intraperitoneally). For echocardiography, mice were induced with 3% isoflurane in an induction chamber and then transferred to an anesthesia mask (Kent Scientific) and imaged under 1.5% isoflurane and continuous O_2_.

### Experimental protocol

After a 1-week acclimation period, healthy mice with an ejection fraction (EF) of ~ 60% were randomly subjected to sham or TAC surgery as described previously [5, 19]. Serial echocardiography was performed to monitor the heart structure and function throughout the course of the experiment. HF was achieved 6 to 8 weeks after the TAC surgery with an ejection fraction of ~ 35%. In this study and our previous study, we used the outbred CD-1 strain [5], which has more genetic variability and is of closer resemblance to clinical applications. Since the CD-1 strain is not the common strain used in cardiac research, we have performed serial echocardiography to ensure that these mice indeed reach HF. Our findings have also have been validated in C57BL/6 mice, the most commonly used strain of mice [5]. We found that the CD-1 strain is more resistant to the banding, thus taking 6–8 weeks to reach HF versus approximately 4 weeks as in the case of the C57BL/6 strain.

Once TAC mice reached HF, they were assigned to one of the following treatment groups for 10 days: a selective ERα-agonist PPT (4,4′,4″-(4-propyl-[1H]-pyrazole-1,3,5-triyl) trisphenol, 850 μg/kg/day, Tocris, PPT group), a selective ERβ-agonist DPN (diarylpropionitrile, 850 μg/kg/day, Tocris, DPN group), and E2 (30 μg E2/kg per day, Innovative Research of America) together with a specific ERβ-antagonist PHTPP (4-[2-phenyl-5,7-bis(trifluoromethyl) pyrazolo[1,5-a]pyrimidin-3-yl]phenol, 850 μg/kg/day, Tocris (E2+ ERβ Antagonist). Dosages of E2, DPN, PPT, and PHTPP were selected based on our prior work [4, 5].

### Cardiac hemodynamics

Serial B-mode and M-mode echocardiography was performed using a VisualSonics (Vevo 2100) equipped with a 30-MHz linear transducer to accurately monitor the stage of the disease by measuring cardiac hemodynamic parameters and heart structure. The EF of control (CTRL) (mice prior to TAC or sham surgery) mice has recently been reported as part of a larger study in the Journal of the American Heart Association [5]. Cardiac hemodynamic parameters were acquired from mechanically ventilated mice under anesthesia (ketamine/xylazine, 100/10 mg/kg, intraperitoneally) via direct left ventricular catheterization in an open-chest procedure by inserting a catheter (1.4F Millar SPR-671) connected to a pressure transducer (Power Lab, ADInstruments) into the LV right before euthanasia. The left ventricular systolic pressure (LVSP), left ventricular end-diastolic pressure (LVEDP), and heart rate (HR) were recorded, and the left ventricular developed pressure (LVDP) and rate pressure product (RPP) were calculated as LVDP = LVSP − LVEDP and RPP = HR × LVDP. The maximum rates of the LV pressure rise (dP/dt_max_) and decline (−dP/dt_min_) were directly calculated from the recordings (Table 1). Hemodynamic data of CTRL (sham-operated mice) and HF mice has also previously been reported by our group [5]. Mice were euthanized by exsanguination under deep anesthesia.

**Table 1:** Estrogen receptor beta activation improves cardiac hemodynamics

	Ctrl (*n* = 10)	TAC (*n* = 7)	ERβ (*n* = 7)	ERα (*n* = 5)
LVSP (mmHg)	104.7 ± 3.9	95.6 ± 5.5	129.4 ± 9.1	86.6 ± 21
LVEDP (mmHg)	6.9 ± 3.8	9.5 ± 4.9	5.2 ± 3.3	32.9 ± 24.0
LVDP (mmHg)	97.8 ± 3.8	86.2 ± 2.7	124.2 ± 7.1*^^^	101.9 ± 8.0
HR	622.8 ± 19.0	421.7 ± 40.4**	520.2 ± 47.1	388.0 ± 22.4**
RPP	60,868.6 ± 2412.8	36,251.9 ± 3531.7*	62,260.7 ± 5963.43^^$^	39,147.7 ± 2905.6*
dP/dt_max_	11,036.6 ± 495.2	4871.4 ± 511.9**	6405.7 ± 641.2**^^^	4777.4 ± 202.8**
dP/dt_min_	8390.9 ± 469.3	4429.2 ± 280.1*	4248.5 ± 1660.6**^^^^	3991.9 ± 196.5**

*P < 0.05 vs. CTRL

**P < 0.001 vs. CTRL

^^^*P* < 0.05 vs. HF

^^^^*P* < 0.001 vs. HF

^$^*P* < 0.05 vs. PPT

### Real-time qPCR

RNA was extracted from whole ventricles snap frozen in liquid nitrogen using the Trizol method (Invitrogen), and cDNA synthesis was achieved using the Omniscript RT kit (Qiagen) according to the manufacturer’s protocol. Real-time qPCR was performed using IQ SYBR Green supermix (BioRad) in a final volume of 25 μL using a BioRad CFX RT-qPCR machine. For all assays, there were at least three samples/group assayed in duplicate. Threshold cycle (Cq value) was determined using CFX Manager, and the Cq value of the gene of interest was normalized to the Cq value of its own internal control gene (GAPDH). Controls consisted of the reaction cocktail without reverse transcriptase and H_2_O instead of cDNA tested by RT-qPCR.

Prior to the RT-qPCR experiments, each primer set was validated to ensure that it yields a single sharp peak in the RT-qPCR melting curve. The controls for each primer set consisted of the reaction cocktail without reverse transcriptase and H_2_O instead of cDNA tested simultaneously by RT-qPCR. All the RT-qPCR products, including the negative controls, were then subjected to gel electrophoresis to ensure amplification of a single product of the expected molecular size without any product amplified in the negative control reactions. Primer sequences were as follows: TGF-β1—forward 5′-CCTGCAAGACCATCGACATGG-3′ and reverse 5′-TGGTTTTCTCATAGATGGCGTT-3′; ANF—forward: 5′-CTGATGGATTTCAAGAACCTGCT-3′ and reverse: 5′-CTCTGGGCTCCAATCCTGTC-3′; collagen I—forward 5′-GACCGATGGATTCCCGTTCGA-3′ and reverse 5′-AAGGTCAGCTGGATAGCGACAT-3′; collagen III—forward 5′-AATTCTGCCACCCCGAACTCAA-3′ and reverse 5′-TCCATCTTGCAGCCTTGGTTAG-3′; fibrosin I (FBRS)—forward 5′-AACACGAACCCTGAGCTGCCA-3′ and reverse 5′-TCATGTAAGCCACACGAACGTG-3′; GAPDH—forward 5′-CCTGCACCACCAACTGCTTAG-3′ and reverse 5′-ATGACCTTGCCCACAGCCTTG-3′; lysil oxydase (LOX)—forward 5′-CACGCAGCAGCAGAATGGGC-3′ and reverse 5′-CGCAGTACCAGCCTCAGCGA-3′; and TGF-β1—forward 5′-CCTGCAAGACCATCGACATGG-3′ and reverse 5′-TGGTTTTCTCATAGATGGCGTT-3′.

### Cell culture

Co-culture of neonatal rat ventricular myocytes (NRVM) and fibroblasts were isolated as previously described [20]. Cells were serum-starved for 24 h and then were serum-replenished and either left untreated (CTRL) or treated with angiotensin II (AngII, 100 nM, Sigma) in the absence or presence of E2 (10 nM), DPN (10 nM), or PPT (10 nM).

### Immunohistochemistry and imaging

Whole hearts were fixed in 4% paraformaldehyde, and transversal 6–7 μm sections were obtained with a cryostat. Tissue sections were used for Masson Trichrome staining (Sigma) according to the manufacturer’s protocol to evaluate fibrosis, and images were acquired with a light microscope (Nikon). For immunofluorescence staining, the heart cross sections (6 μm) were labeled with anti-CD-31 antibody (Millipore (04-1074), 1:200 dilution) and wheat germ agglutinin (WGA, Invitrogen, 1:500 dilution), and images were acquired with a confocal scanning microscope (Nikon). Quantification of angiogenesis and fibrosis was performed using ImageJ.

### Statistical analysis

One-way ANOVA with Holm-Sidak post hoc tests were used to compare between groups. *P* values less than 0.05 were considered statistically significant. Values are expressed as mean ± SEM.

## Results

### Estrogen improves cardiac contractility in HF mice mainly via estrogen receptor beta

**Fig. 1:**
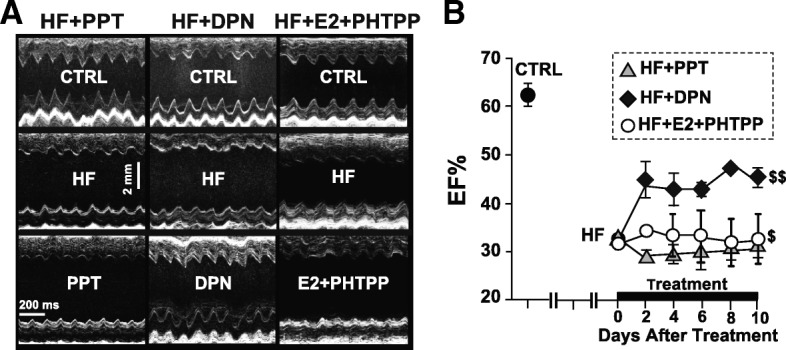
ERβ-agonist, but not ERα-agonist, significantly improves HF. **a** Examples of M-mode images of the parasternal short axis view by echocardiography before surgery (CTRL), in heart failure (HF) and 10 days after treatment with an ERα-agonist (PPT, left panels), ERβ-agonist (DPN, center panels), or E2 together with the ERβ-specific antagonist PHTPP (right panels). **b** Averaged EF as a function of time in PPT (triangles, *n* = 6), DPN (diamonds, *n* = 7), and E2+PHTPP (circles, *n* = 4). ^$^*P* < 0.05 vs. PPT and ^$$^*P* < 0.001 vs. PPT

Recently, we reported that E2 rescues pre-existing HF in mice by restoring the ejection fraction (EF) of HF mice from 33.2 ± 1.1% in HF to 53.1 ± 1.3% within 10 days of E2 treatment. We have further showed that this protection is conferred on C57BL/6 mice to a similar degree, as their EF after 10 days of E2 treatment was 56.24 ± 2.40% [5]. Most of the biological actions of E2 are mediated through ERα or ERβ, and both of these receptors are present in the heart [8]. Here, we examined the role of ERα and ERβ in the rescue action of E2 against HF. Serial echocardiography revealed that the EF of mice treated with the ERβ-agonist DPN significantly improved (from 33.2 ± 1.2% to 45.3 ± 2.1%, *n* = 7), while there was no improvement in the EF of HF mice treated with ERα-agonist PPT (from 33.0 ± 1.5% to 31.1 ± 2.3%, *n* = 6, Fig. 1). Similarly, fractional shortening improved only in DPN-treated mice from 15.8 ± 0.6% to 21.9 ± 1.6% in DPN (*P* < 0.001, *n* = 7) vs. from 15.8 ± 0.8% to 14.7 ± 1.2% in PPT (*n* = 6). To further investigate the involvement of ERβ in the rescue action of E2, some HF mice were treated with E2 in the presence of the ERβ-antagonist PHTPP. E2 failed to rescue HF in the presence of PHTPP, as there was no significant improvement in their EF at the end of 10-day treatment (from 31.5 ± 1.1% to 32.5 ± 5.2%, *n* = 4, Fig. 1a, b).

### Anti-hypertrophic action of E2 is mediated through ERβ

**Fig. 2:**
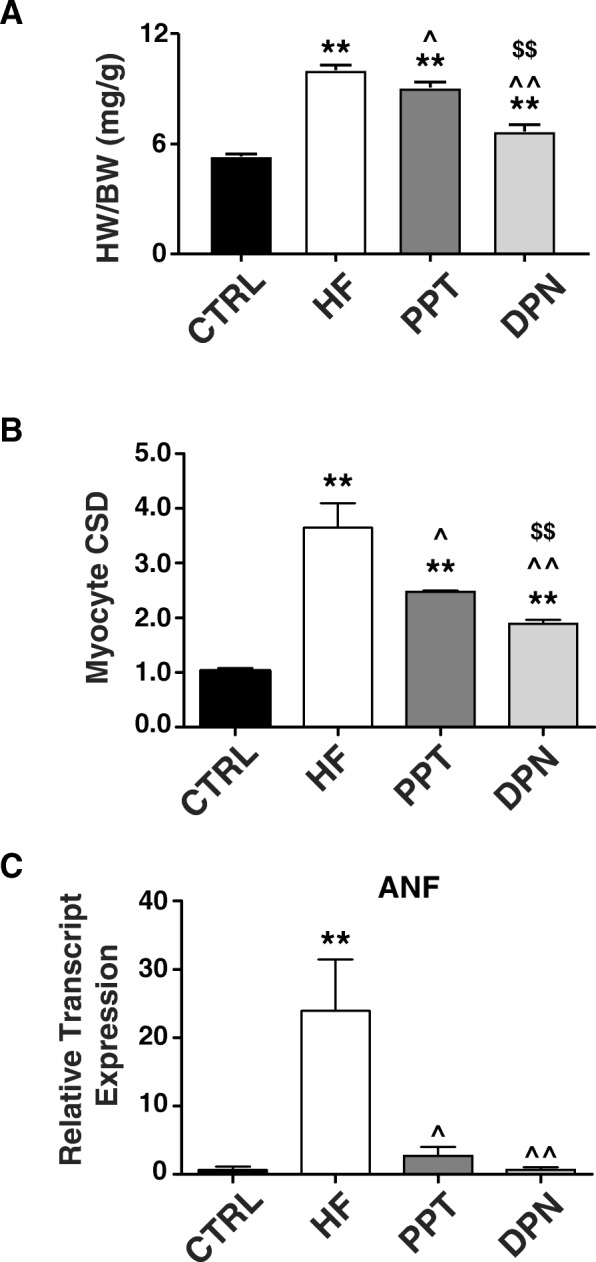
ERβ activation is associated with reduced cardiac hypertrophy and decreased expression levels of fetal gene transcripts. **a** Heart weight to body weight ratio (HW/BW) in mg/g. **b** Myocyte cross-sectional diameter (CSD) normalized to CTRL. **c** Relative transcript expression normalized to CTRL of ANF in CTRL (sham-operated animals), HF, after PPT, or DPN treatment (*n* = 3–6 mice/group). *P < 0.05 vs. CTRL, **P < 0.001 vs. CTRL, ^^^*P* < 0.05 vs. HF, ^^^^*P* < 0.001 vs. HF, and ^$$^*P* < 0.001 vs. PPT

To examine the effect of PPT and DPN on cardiac hypertrophy, heart weight to body weight (HW/BW) ratio was first measured. Treatment with DPN was associated with decreased cardiac hypertrophy as demonstrated by a significantly decreased HW/BW ratio to 6.84 ± 0.35 from 10.15 ± 0.30 in HF (Fig. 2a), while PPT had no effect as the HW/BW of the PPT-treated animals was not different than that of the HF group (9.17 ± 0.32). The decreased in HW/BW ratio with DPN was mainly due to the reduction in myocyte cross-sectional diameter (CSD) as DPN administration was able to drastically reduce the CSD from 3.01 ± 0.40 in HF to 1.80 ± 0.16 (Fig. 2b), while PPT had only partial effect in reducing CSD to 2.37 ± 0.13. While the ERα agonist PPT had an effect in downregulating ANF transcript levels, DPN significantly restored ANF transcripts to levels similar to CTRL (from 24.27 ± 6.25 in HF to 0.84 ± 0.16 with DPN, Fig. 2c).

### Estrogen attenuates cardiac fibrosis in HF mainly via estrogen receptor beta

**Fig. 3:**
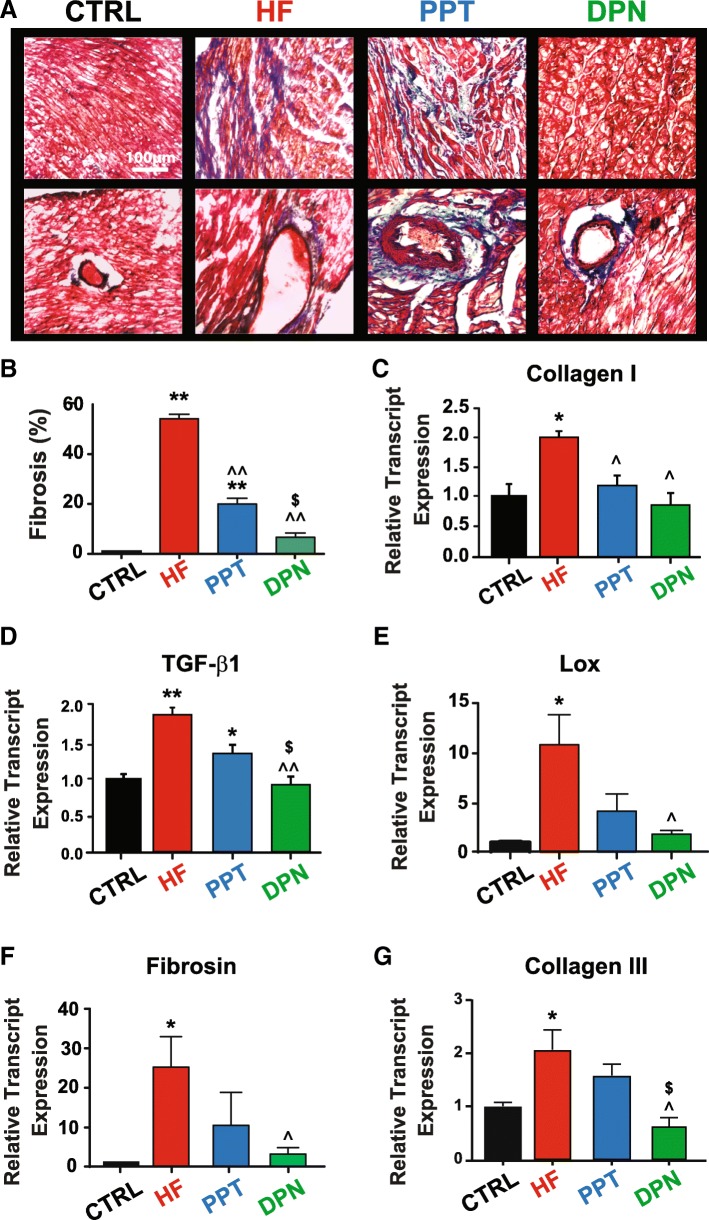
ERβ-agonist DPN attenuates both interstitial and perivascular cardiac fibrosis in HF mice. **a** Representative Masson’s Trichrome staining of LV sections (interstitial, top panels; perivascular, lower panels) in HF mice treated with the ERα-agonist PPT or the ERβ-agonist DPN for 10 days; blue color indicates fibrosis. **b** Quantification of overall fibrosis expressed as percentage of each high-power field. **c**–**g** Relative transcript expression of in vivo of profibrotic genes normalized to CTRL (sham-operated mice). GAPDH was used as an internal control (*n* = 3–6 mice/group). *P < 0.05 vs. CTRL, **P < 0.001 vs. CTRL, ^^^*P* < 0.05 vs. HF, ^^^^*P* < 0.001 vs. HF, ^$^*P* < 0.05 vs. DPN, and ^$$^*P* < 0.001 vs. PPT

We recently reported that E2-induced rescue of HF is associated with reversal of cardiac fibrosis [5]. Here, we examined the role of ERα and ERβ activation in cardiac fibrosis on mice with pre-existing HF. We found that DPN therapy attenuates both interstitial and perivascular fibrosis, while the ERα-specific agonist PPT is not as effective (Fig. 3a, b). As previously reported, HF was associated with widespread cardiac fibrosis. Here, we report that the ERβ agonist was able to significantly attenuate fibrotic scarring to 6.67 ± 1.76% from 54.25 ± 1.80% observed in HF, while PPT had a partial effect (20.00 ± 2.31%). To investigate the mechanism through which DPN can exert its beneficial actions on attenuating fibrosis, we assessed the transcript levels of several fibrotic markers. RT-qPCR revealed that the transcript levels of several profibrotic markers were significantly upregulated in HF (from 1.00 ± 0.29 to 2.03 ± 0.05 for collagen I; 1.00 ± 0.07 to 2.05 ± 0.36 for collagen III; 1.00 ± 0.07 to 1.87 ± 0.12 for TGF-β1; 1.0 ± 0.5 to 3.6 ± 0.2 for fibrosin I and 1.0 ± 0.08 to 10.59 ± 2.84 for LOX), and DPN treatment significantly restored all these transcripts to levels comparable to the CTRL (sham-operated) group (Fig. 3c–g).

**Fig. 4:**
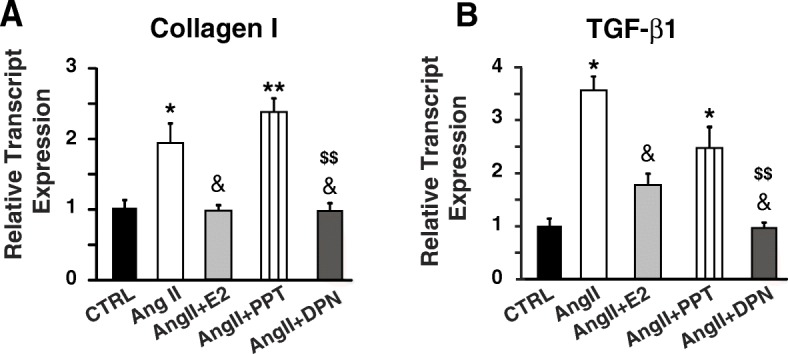
DPN reverses AngII-induced upregulation of collagen I and TGF-β1 transcripts in vitro. Relative transcript expression of in vitro collagen I (**a**) and TGF-β1 (**b**). *N* = 3–5 individual treatments/group. *P < 0.05 vs. CTRL, **P < 0.001 vs. CTRL, ^&^*P* < 0.05 vs. AngII, and ^$$^*P* < 0.001 vs. AngII+DPN

Consistent with our in vivo findings, E2 and DPN treatments were able to reverse AngII-induced transcript upregulation of collagen I and TGF-β1 in a co-culture of neonatal rat fibroblasts and ventricular myocytes, while PPT was only partially effective in reducing the levels of TGF-β1 but had no effect on collagen I expression (Fig. 4a, b).

### Estrogen receptor beta, but not alpha, activation is associated with increased cardiac angiogenesis in HF mice

**Fig. 5:**
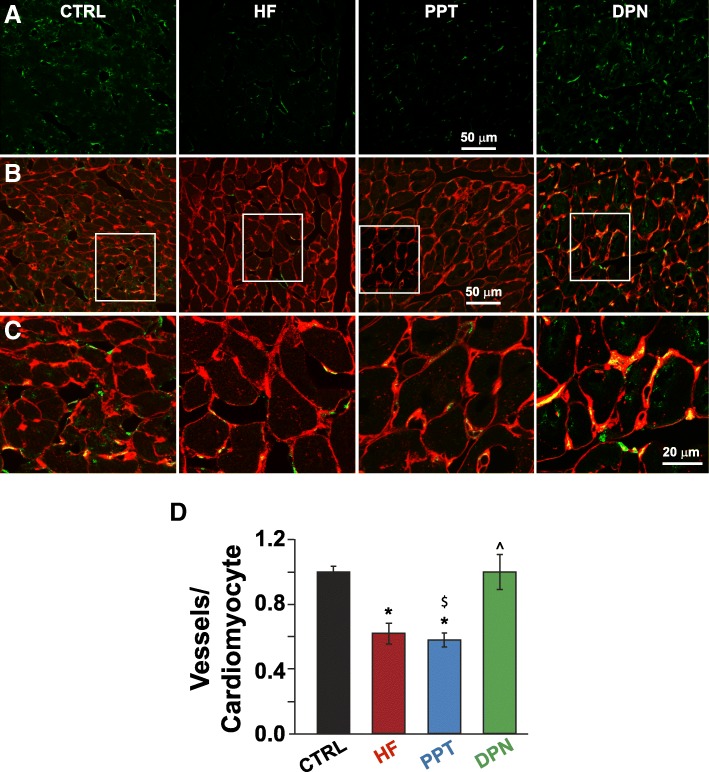
Treatment of HF mice with the ERβ agonist DPN is associated with increased cardiac angiogenesis in HF mice. Confocal images of LV sections immunostained for CD-31 (**a**), the overlay of CD-31 and WGA (**b**), and at higher display magnification of the white squares (**c**). **d** Quantification of capillary density as microvessels per cardiomyocyte. CTRL group was comprised of sham operated mice *P < 0.05 vs. CTRL, ^^^*P* < 0.05 vs. HF, and ^$^*P* < 0.05 vs. PPT (*n* = 3–4 animals per group)

Estrogen has been known to promote angiogenesis in several tissues, and recently, we have shown that it promotes new blood vessel formation in the heart as well. We showed that E2 therapy enhanced capillary density in HF mice and that in the presence of an angiogenesis inhibitor, E2 could not exert its beneficial action on rescuing the heart from failure [5]. Here, we examined the role of ERα and ERβ in stimulating angiogenesis by E2 in male mice with HF. We have performed immunostaining of cardiac cross sections with anti-cluster of differentiation-31 (CD-31, also known as platelet endothelial cell adhesion molecule-1) antibody, in order to determine new blood vessel formation. We found that the capillary density, measured as microvessels per cardiomyocyte, of HF mice treated with the ERβ-agonist DPN was similar to that of CTRL (sham-operated mice, 1 ± 0.1 in DPN vs. 0.62 ± 0.06 in HF and 1 ± 0.03 in CTRL, Fig. 5), while there was no difference in capillary density between the ERα-agonist PPT and the HF group (0.58 ± 0.04, Fig. 5b).

### Estrogen receptor beta stimulation improves cardiac hemodynamic parameters in HF mice

**Fig. 6:**
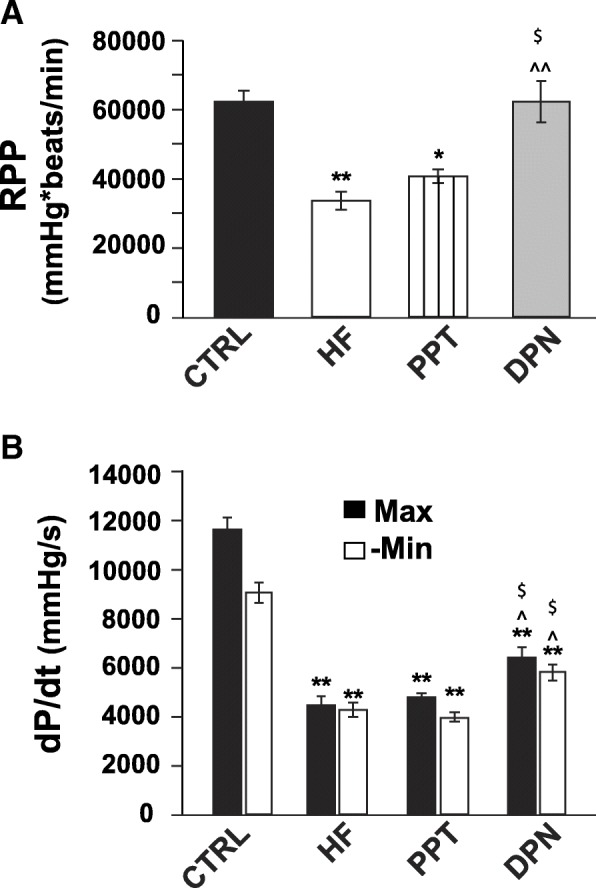
ERβ-agonist therapy is associated with improved systolic function and attenuation of contraction and relaxation defects induced by HF. **a** RPP was determined right before euthanasia by direct LV catheterization and was calculated as HR × LVDP where LVDP = LVSP − LVEDP. **b** dP/dt_Max_ (maximum rate of the LV pressure rise, filled bars) and −dP/dt_Min_ (maximum rate of the LV decline) were directly calculated from the recordings. CTRL group consisted of sham-operated mice. *P < 0.05 vs. CTRL, **P < 0.001 vs. CTRL, ***P < 0.0001 vs. CTRL, ^^^*P* < 0.05 vs. HF, ^^^^*P* < 0.001 vs. HF, and ^$^*P* < 0.05 vs. PPT (*n* = 5–7 animals/group)

We have recently shown that E2-induced rescue of HF was associated with improved mechanical performance of the heart, since the rate pressure product (RPP) was significantly increased in the group of mice which received 10 days of E2 therapy compared to HF [5]. The cardiac contraction and relaxation defects induced by HF were also corrected by E2 therapy, as the maximum rates of LV pressure rise (dP/dt_max_) and decline (dP/dt_min_) were attenuated with E2 treatment to values similar to healthy hearts in spite of the sustained presence of the TAC stress stimulus. Here, we examined the role of ERα and ERβ in E2-induced improvement of hemodynamic parameters in HF mice. Treatment with the ERβ-agonist DPN was associated with improved cardiac hemodynamics, as the RPP and LVDP of mice in the DPN-treated group were significantly improved to 62,261 ± 5963 from 33,530 ± 2663 for the RPP and to 124.2 ± 7.1 from 86.2 ± 2.7 for the LVDP versus HF, while PPT treatment had no effect on LV mechanical performance as the RPP (40,666 ± 2020) and LVDP (101.9 ± 8.0) were not significantly different from HF (Fig. 6a, Table 1). DPN treatment was also associated with an attenuation of the relaxation and contraction defects induced by HF, as the dP/dt_max_ and dP/dt_min_ were also significantly higher following DPN treatment compared to HF (dP/dt_max_ from 4871.4 ± 511.9 mmHg/s to 6406 ± 461, and −dP/dt_min_ from 4298 ± 293 mmHg/s to 5829 ± 322), while PPT had no significant effect on these parameters (dP/dt_max_ 4777 ± 203 mmHg/s, and −dP/dt_min_ from 3992 ± 196 mmHg/s (Fig. 6b, Table 1).

## Discussion

We have recently shown that exogenous E2 therapy starting after the onset of HF rescues HF in male mice (CD-1 as well as C57BL/6) [5]. Here, we examined the contribution of ERα and ERβ in E2-induced rescue of HF. Our data using specific ERα and ERβ agonists clearly demonstrated that ERβ activation is associated with the salutary effects of E2 in the rescue of HF (Fig. 1). The ERβ-agonist DPN significantly improved EF of HF mice, while there was no improvement in the EF of HF mice treated with the ERα-agonist PPT (Fig. 1). Furthermore, the fact that E2 failed to rescue HF in the presence of a specific ERβ-antagonist further confirmed the role of ERβ in the E2-induced cardioprotection against pressure overload (Fig. 1). Here we also show that ERβ activation is anti-hypertrophic, as DPN-treated mice have lower HW/BW ratio, myocyte CSD, and restored ANF transcript expression when compared to HF and PPT-treated mice (Fig. 2). We show that the anti-fibrotic action of E2 in the failing heart is mediated mainly through ERβ, as treatment with the ERβ agonist DPN attenuated fibrosis and restored the expression of profibrotic gene transcripts back to CTRL (sham) levels, while the ERα agonist PPT had a minimal effect (Fig. 3). In vitro, DPN fully restored the transcript levels of the fibrotic markers collagen I and TGF-β1 to CTRL levels, while PPT had no effect at all (Fig. 4). We also demonstrated that the improvement of cardiac function of HF mice treated with DPN, but not PPT, was also associated with increased cardiac angiogenesis (Fig. 5). Lastly, here we show that ERβ activation was associated with improved hemodynamic parameters, and attenuated contractile defects whereas ERα activation had no significant effect (Fig. 6, Table 1).

### Role of estrogen receptors in heart function

Both classical estrogen receptors ERα and ERβ as well as the newly identified E2 receptor G protein coupled receptor 30 (GPR30) are all expressed in the heart [5, 7, 8, 21]. Among these three E2 receptors, the beneficial role of ERβ in cardioprotection has been well documented in the recent years [4, 9, 10]. Female mice lacking the ERβ gene are less protected against myocardial ischemia/reperfusion (I/R) injury compared to wild-type female mice [22]. Long-term (2 weeks) pre-treatment with the ERβ-agonist DPN protects the heart against I/R injury in ovariectomized female mice possibly due to upregulation of cardioprotective genes, such as those encoding nitric oxide (NO) biosynthesis and antiapoptotic proteins [9]. Chronic DPN treatment in OVX mice leads to activation of protein S-nitrosylation and cardioprotection, which was blocked by nitric oxide synthase (NOS) inhibition [23].

ERβ knockout OVX female mice have abnormal vascular function and hypertension, increased mortality, and aggravated heart failure [11]. ERβ is also shown to be responsible for the anti-hypertrophic and anti-fibrotic effects of E2 in OVX female (22), intact male and female (27), and intact male mice [10, 24, 25]. Our data using specific ERα and ERβ agonists clearly demonstrates that ERβ is the primary receptor responsible for the salutary effects of E2 in the rescue of HF. Additionally, the fact that E2 failed to rescue HF in the presence of a specific ERβ-antagonist further confirms the role of ERβ in E2-mediated rescue of pressure overload-induced HF. Similarly, our group has also reported that the protective action of E2 in intact male rats in the context of right ventricular failure secondary to pulmonary hypertension (PH) is also mainly mediated through ERβ [4].

Regarding the role of ERα in the heart, acute activation of ERα seems to protect the heart from ischemic injury, while the data is conflicting regarding the role of ERα under conditions of chronic E2 exposure [26]. Booth et al. showed that acute pre-treatment with E2 or PPT, but not DPN, significantly decreased infarct size in an in vivo OVX female rabbit model of ischemia/reperfusion injury [27]. Recently, Westphal et al. demonstrated that specific ERα activation prevents loss of systolic function in ovariectomized female mice subjected to TAC surgery [17]. Our data, however, showed no significant improvement in the EF or hemodynamic parameters of HF mice with PPT treatment (Figs. 1 and 6, Table 1). This discrepancy could be attributed to the different protocol used. Indeed, Westphal et al. used a prevention protocol whereas we used a reversal protocol [17]. Thus, it is possible that ERα activation is not able to reverse pre-existing HF but can prevent the development of HF.

In our experimental setting, although ERβ activation significantly increases the EF of HF mice from 33 to 45%, it is not as efficient as E2 in the reversal of pre-established HF. This difference can be attributed to the activation of GPR30, as GPR30 has recently been demonstrated to protect the heart against I/R injury through inhibition of mitochondrial transition pore opening [21]. In addition, in vivo activation of GPR30 attenuates diastolic dysfunction and fibrotic deposition associated with the loss of estrogen in oophorectomized mRen2.Lewis rats [28].

### Role of estrogen receptors in cardiac fibrosis

We have recently shown that E2-induced rescue of HF in intact male mice is associated with reversal of LV fibrosis by reducing the expression of several pro-fibrotic genes [5]. Lee et al. investigated the expression of the classic ERs in fibroblasts and found that both ERα and ERβ were expressed, with the predominant receptor being ERβ expressed both in the cytosol and nucleus [29]. Recently, growing evidence demonstrates a distinct role of estrogen receptors on cardiac fibrosis. For example, ERα overexpression in male mice does not prevent development of cardiac fibrosis [30]. However, ERβ overexpression decreases JNK phosphorylation and protects intact male and female mouse hearts against fibrosis [16]. Furthermore, a recent study highlighted the fact that ERα activation in fibroblasts increased fibronectin production [31]. On the other hand, in male mice, ERβ has been demonstrated to have a beneficial role on cardiac fibrosis, at least in part by inhibiting TGF-β1 and collagen I expression in intact male and female mice (13) and OVX female mice [10, 18]. ERβ is also known to block the transition of fibroblasts into myofibroblasts. ERβ-specific activation has been shown to block cardiac fibrosis by decreasing SMAD3, the downstream target of TGF-β1 and AngII, which facilitates the transition of fibroblasts to myofibroblasts and thus reduces overall fibrotic deposition. The same study also demonstrated that E2 prevented hypertrophy induced by AngII infusion in WT mice, while E2 could not exert any beneficial effects in ERβ knockout OVX female mice [10]. This data provides further evidence for the anti-fibrotic and anti-hypertrophic role of ERβ.

Here, we also show that the anti-fibrotic action of estrogen in the failing heart is mediated mainly through ERβ, as treatment with the ERβ-agonist DPN decreased fibrosis, as well as effectively restored the levels of the fibrotic markers collagen I and TGF-β1 to levels similar to control in vivo. While the ERα-agonist PPT had a minimal effect in the reversal of fibrosis in the heart and was able to decrease the transcript levels of collagen I and TGF-β1, it was not able to significantly decrease the elevated transcript levels of collagen III, LOX and fibrosin I observed in HF. In vitro, DPN fully restored the transcript levels of collagen I and TGF-β1 to CTRL levels, while PPT had no effect at all. The discrepancy observed between our in vivo and in vitro data could be due to the fact that in the working myocardium, we observe the contribution and effect of other cell types and not just fibroblasts.

### Role of E2 receptors in angiogenesis

HF can be attributed, at least in part, to the imbalance between myocardial oxygen consumption and supply that will drive the switch from cardiac hypertrophy to cardiac decompensation and HF [12]. Thus, improving the blood supply by activating angiogenesis is a critical aspect in preventing and rescuing HF. E2 has been shown to be pro-angiogenic in various tissues and organs such as the uterus, breast, brain, and limbs [12–14]. We have recently shown that E2 could also stimulate angiogenesis in two different models of HF, in LV hypertrophy induced by pressure overload as well as in right ventricular (RV) hypertrophy induced by PH in intact male and female mice as well as male rats [4, 5].

The cardioprotective action of ERβ is also attributed in part to stimulation of angiogenesis through activation of VEGF, basic fibroblast growth factor (bFGF), and cyclo-oxygenase 2 (COX2). Activation of ERβ by E2 also leads to increased expression of NOS [32], a well-known powerful vasodilator, which is also required for angiogenic properties of vascular endothelial growth factor (VEGF) [33]. Indeed, in endothelial cell cultures in the presence of NO antagonist, VEGF failed to promote angiogenesis [33]. This effect of ERβ on angiogenesis is reinforced by a positive feedback loop where the prostanoids synthetized by COX2 promote VEGF and fibroblast growth factor (FGF) expression [34].

Here, we show that, ERβ, but not ERα activation, improves cardiac functional parameters concomitantly with reducing fibrosis and normalizing blood vessel formation to levels similar to healthy control mice (Fig. 5). Although ERβ activation was able to normalize angiogenesis to levels comparable to CTRL, it was not as potent as E2 which upregulated angiogenesis ~ 3-fold above control levels. This could partially account for the lower EF observed in HF mice treated with DPN compared to E2 (from ~ 45% in DPN vs. ~ 55% in E2, as we previously reported [5]). The difference between stimulation of angiogenesis between E2 and DPN could be due to the activation of GPR30. Indeed, GPR30 is also known to promote angiogenesis by activating VEGF [35].

## Conclusions

We have recently shown that E2 rescues pre-existing HF by improving EF from ~ 35 to 55% through stimulating cardiac angiogenesis and reversing cardiac fibrosis in intact male mice [5]. Here, we demonstrate that ERβ activation is sufficient to decrease cardiac fibrosis and restore angiogenesis, as well as to significantly improve cardiac hemodynamic parameters. Selective agonists for ERβ such as DPN do not stimulate breast and ovarian cancer growth and therefore offer a safer alternative for therapeutic intervention to minimize the unwanted side effects of E2. Our data highlights the potential clinical interest of ERβ-specific agonists for the treatment of chronic heart failure in humans within a relatively safe time frame, which certainly is a concept that warrants further investigation.

## Abbreviations

ANF: Atrial natriuretic factor
AngII: Angiotensin II
bFGF: Basic fibroblast growth factor
CD-31: Cluster of differentiation 31
COX2: Cyclo-oxygenase 2
CSD: Cross-sectional diameter
CTRL: Control
dP/dt_max_: Maximum rate of the LV pressure rise
dP/dt_min_: Maximum rate of the LV pressure decline
DPN: Diarylpropionitrile
E2: Estrogen
EF: Ejection fraction
ERα: Estrogen receptor alpha
ERβ: Estrogen receptor beta
FGF: Fibroblast growth factor
GPR30: G protein-coupled receptor 30
HF: Heart failure
HR: Heart rate
HW/BW: Heart weight to body weight
I/R: Ischemia/ reperfusion
LOX: Lysil oxidase
LV: Left ventricular
LVEDP: Left ventricular end-diastolic pressure
LVSP: Left ventricular systolic pressure
NO: Nitric oxide
NOS: Nitric oxide synthase
NRVM: Neonatal rat ventricular myocytes
PH: Pulmonary hypertension
PHTPP: 4-[2-Phenyl-5,7-bis(trifluoromethyl)pyrazolo[1,5-a]pyrimidin-3-yl]phenol
PPT: 4,4′,4″-(4-Propyl-[1H]-pyrazole-1,3,5-triyl) trisphenol
RPP: Rate pressure product
RV: Right ventricular
SMAD3: Mothers against decapentaplegic homolog 3
TAC: Transverse aortic constriction
TGF-β1: Transforming growth factor-β1
VEGF: Vascular edothelial growth factor
WGA: Wheat germ agglutinin
WT: Wild-type
